# Whole-Genome Shotgun Sequencing of *Mycobacterium abscessus* M156, an Emerging Clinical Pathogen in Malaysia

**DOI:** 10.1128/genomeA.00063-12

**Published:** 2013-01-15

**Authors:** Siew Woh Choo, Yan Ling Wong, Ching Yew Beh, Naline Lokanathan, Mee Lian Leong, Chia Sui Ong, Kee Peng Ng, Yun Fong Ngeow

**Affiliations:** a Department of Medical Microbiology, Faculty of Medicine, University of Malaya, Kuala Lumpur, Malaysia; b Dental Research and Training Unit, Faculty of Dentistry, University of Malaya, Kuala Lumpur, Malaysia; c Faculty of Information Science and Technology, Multimedia University, Melaka, Malaysia

## Abstract

*Mycobacterium abscessus* is an emerging clinical pathogen commonly associated with non-tuberculous mycobacterial infections. We report herein the draft genome of *M. abscessus* strain M156.

## GENOME ANNOUNCEMENT

*Mycobacterium abscessus* has been considered the most pathogenic and chemotherapy-resistant rapid-growing mycobacterium. It is an environmental organism commonly associated with a wide range of clinical diseases such as pulmonary and skin and soft tissue infections (1) which can develop into fatal, deep-seated, and disseminated diseases. Treating *M. abscessus* infections can be difficult due to its resistance to most of the antimycobacterial agents (2). The genomic composition of the organism could provide valuable insights into its pathogenicity, antimicrobial resistance, and virulence in the human host.

Here we report the draft genome of *M. abscessus* strain M156 isolated from the sputum sample of a Malaysian male chronic smoker presenting with bronchiectasis and hemoptysis. Whole-genome shotgun sequencing of strain M156 using the Illumina genome analyzer 2X technology generated a total of 3,937,129 reads. The M156 draft genome was 5,021,782 bp in length with a high GC content of 64.1%. Sequence assembly with a Genomics workbench 4.9 resulted in 69 contigs with an N_50_ of 221,929 bp. Contigs were autoannotated with Rapid Annotation Subsystems Technology (RAST) server (3). A total of 5,087 open reading frames (ORFs) were identified, with 45 tRNA and 3 rRNA encoding genes. The M156 draft genome has an average coverage of 87.5% and an average identity of 97.3% in comparison with the reference genome *M. abscessus* ATCC 19977 (4).

Automated annotation revealed that the genome may contain 5,039 predicted coding sequences, of which 3,491 (69%) are involved in the subsystems. A total of 433 genes are responsible for encoding amino acids and derivatives, and 391 genes for encoding cofactors, vitamins, prosthetic groups, and pigments. The pathogenicity of the organism could be linked to 41 genes encoding proteins predicted to be associated with virulence, disease, and defense and 28 genes specifically linked to phage and prophage elements. Further studies on these putative proteins may increase our understanding of *M. abscessus* human infections.

### Nucleotide sequence accession number. 

The *M. abscessus* strain M156 genome sequence and its annotations have been deposited in NCBI GenBank under the accession number AKVU00000000.
